# Supplementary material for: Luteolin Induces Selective Cell Death of Human Pluripotent Stem Cells
Source: Biomedicines. 2020 Oct 27;8(11):453. doi: 10.3390/biomedicines8110453 (PMC7692041; doi:10.3390/biomedicines8110453)
Supplement: Supplementary file 1 [file biomedicines-08-00453-s001.zip › biomedicines-964418 supplementary v2/biomedicines-964418 supplementary.v2.pdf]

Supplementary materials

Supplementary Figure legends

Figure S1 (A) mRNA expression of indicated gene in hESCs (H9) and hDFs (B) Graphical presentation of live cells (dual negative population of Annexin V/7-AAD staining) at 24 hours after treatment of 50μM of each flavonoid in hESCs

Figure S2 IC50 plot of LUT and QC

Movie S1. Real time images of hESCs after treatment of mock (A) QC (B) or LUT (C)

Movie S2. Real time image of spontaneous contraction in hESC-CMs

Movie S3. Real time fluorescence image of Ca^{2+} oscillation in hESC-CMs

Movie S4. Real time fluorescence image of Ca^{2+} oscillation in hESC-CMs after treatment of Mock (A) or 12.5μM of LUT (B)

Figure. S1

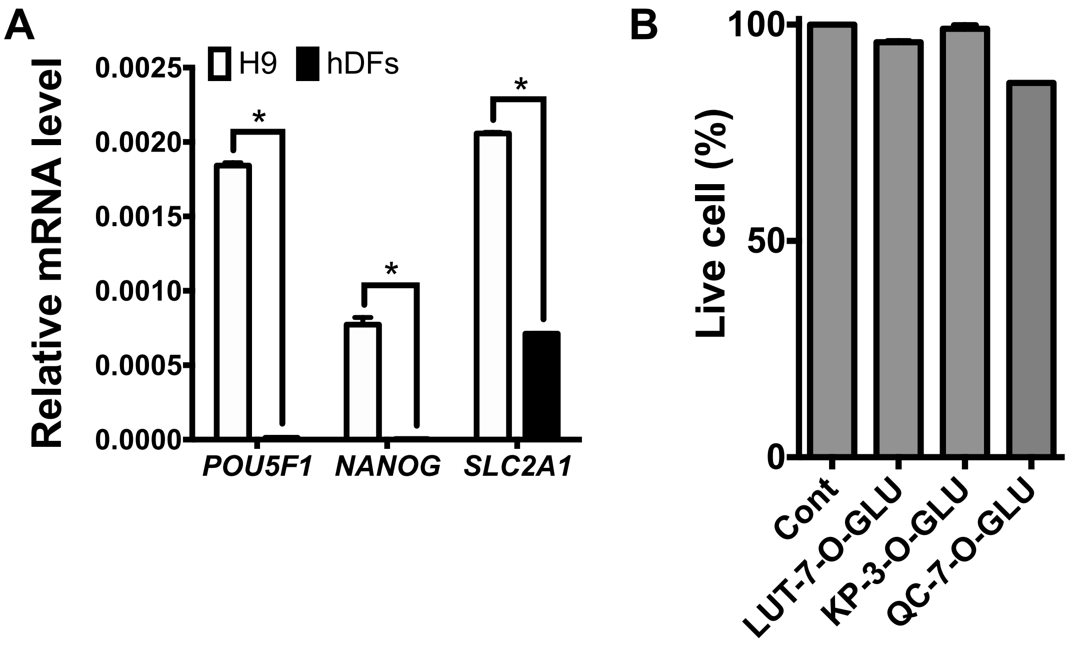

A
Relative mRNA level
0.0025
0.0020
0.0015
0.0010
0.0005
0.0000
H9
hDFs
*
*
*
POU5F1
NANOG
SLC2A1
B
Live cell (%)
100
50
0
Cont
LUT-7-O-GLU
KP-3-O-GLU
QC-7-O-GLU

Figure. S2

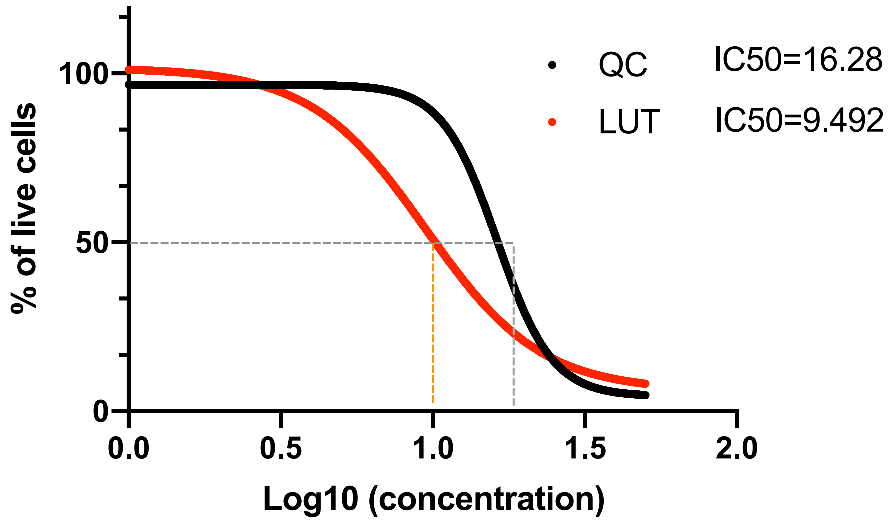
100
50
0
% of live cells
0.0
0.5
1.0
1.5
2.0
Log10 (concentration)
QC
IC50=16.28
LUT
IC50=9.492
